# Mitral Transcatheter Edge-to-Edge Repair in Acute Ischemic Mitral Regurgitation: Current Evidence and Future Perspectives

**DOI:** 10.31083/RCM33396

**Published:** 2025-04-21

**Authors:** Marco Frazzetto, Claudio Sanfilippo, Francesco Briguglio, Chiara Giacalone, Claudia Contrafatto, Andrea Munafò, Michela Bonanni, Jacopo Oreglia, Giuliano Costa, Guilherme Attizzani, Davide Capodanno, Carmelo Grasso

**Affiliations:** ^1^Harrington Heart & Vascular Institute, University Hospitals, School of Medicine, Case Western Reserve University, Cleveland, OH 44106, USA; ^2^De Gasperis Cardio Center, Interventional Cardiology Unit, Niguarda Hospital, 20162 Milan, Italy; ^3^Division of Cardiology, A.O.U. Policlinico “G. Rodolico - San Marco”, 95123 Catania, Italy; ^4^Department of Cardiology, Policlinico Tor Vergata, University of Rome, 00133 Rome, Italy

**Keywords:** acute mitral regurgitation, mitral transcatheter edge-to-edge repair, Mitraclip

## Abstract

Acute ischemic mitral regurgitation is a rare but potentially catastrophic complication following acute myocardial infarction (AMI), characterized by severe clinical presentation and high mortality. Meanwhile, advancements in primary percutaneous coronary intervention (PCI) have reduced the incidence of acute mitral regurgitation (AMR). The surgical approach remains the standard treatment but is associated with high rates of complications and in-hospital mortality, particularly in patients with cardiogenic shock or mechanical complications, such as papillary muscle rupture. Mitral transcatheter edge-to-edge repair (M-TEER) has emerged as a minimally invasive treatment. Current evidence demonstrates the feasibility and safety of M-TEER in reducing mitral regurgitation, stabilizing hemodynamics, and improving in-hospital and short-term survival. The procedural success rate is high, with notable symptoms and functional status improvements. Mortality rates remain significant, reflecting the severity of AMR, but are lower compared to medical management alone. Challenges remain regarding the optimal timing of M-TEER, long-term device durability, and patient selection criteria. Ongoing iterations in device technology and procedural techniques are expected to enhance outcomes. This review highlights the role of M-TEER in AMR management, emphasizing the need for multidisciplinary decision-making and further research to refine M-TEER application and improve outcomes in this high-risk AMR population.

## 1. Introduction

Mitral regurgitation (MR) is a common finding following acute myocardial infarction (AMI) even in the primary percutaneous coronary intervention (PCI) era, negatively impacting early outcomes and long-term survival [1, 2]. Acute ischemic severe MR is a life-threatening and potentially catastrophic condition. However, as much as severe acute mitral regurgitation (AMR) is typically associated with symptoms and heart failure, the clinical spectrum of presentation is varied and dependent on several factors [3]. Two distinct phenotypes of ischemic AMR are generally described. The first etiology is associated with complete or partial papillary muscle rupture (or primary), whilst the functional (or secondary) type results from a combination of systolic leaflets tethering and acute regional wall abnormalities. Regardless of the phenotype presentation, the sudden onset of massive regurgitant volume into a normal compliant left atrium (LA) markedly increases LA pressure and affects pulmonary venous pressure leading to pulmonary edema. In the attempt to preserve stroke volume, there is a marginal degree of initial compensation by increasing preload, but LA and left ventricle (LV) cannot accommodate the incremented volume which leads to increased LV end-diastolic and LA pressures and forward LV failure [3]. Recognizing the potential variation in clinical symptoms presentation, the recent proposal of a clinical classification for AMR, which distinguishes 4 different subtypes, is an essential tool to perform risk stratification from the diagnosis and improve outcomes [4]. In “Type 1”, patients exhibit the most severe symptoms, including cardiogenic shock (CS), pulmonary edema, and possible cardiac arrest. In “Type 2”, patients have severe heart failure with pulmonary edema and maintain blood pressure but potentially cardiac output can be reduced. “Type 3” is less severe, characterized by recurrent pulmonary congestion and sudden pulmonary edema. Patients with severe MR and mild to moderate symptoms of heart failure are included in the “Type 4” [4, 5]. The first three types are susceptible to emergent transcatheter or surgical treatment given the critical condition.

The optimal management of patients with AMR is uncertain because of the lack of randomized clinical trials in this setting. Primary PCI is paramount to prevent and potentially reduce severe AMR [6]. Medical and supportive management is demanding and can ameliorate the severity of the symptoms caused by volume overload, but a significant portion of this population continues to experience recurrent pulmonary edema and low cardiac output and thus requires an urgent evaluation for immediate intervention [6]. Surgical treatment, in this context, is the first choice but it is associated with higher rates of mortality and a large portion of this population is often deemed at prohibitive surgical risk and medically managed [6]. In the last decade, the introduction and diffusion of mitral transcatheter edge-to-edge repair (M-TEER) has offered an alternative option for these patients. This review aims to discuss the latter advancements in the management of patients with ischemic AMR, focusing on the current evidence of the M-TEER in this population.

## 2. Mechanism of Ischemic Acute Mitral Regurgitation

### 2.1 Primary Acute Ischemic Mitral Regurgitation

The leading cause of MR following AMI is papillary muscle rupture (PMR). While complete rupture occurs in just 1%–3% of AMI cases, it typically causes severe illness, rapid deterioration, and poor survival rates.

AMR resulting from a complete or partial PMR represents a rare but critical complication of AMI. Over recent decades, its incidence has decreased, largely due to advancements in revascularization strategies. Nonetheless, the in-hospital mortality rate associated with post-AMI MR remains alarmingly high, ranging from 36% to 80% [7].

**Fig. 1.**
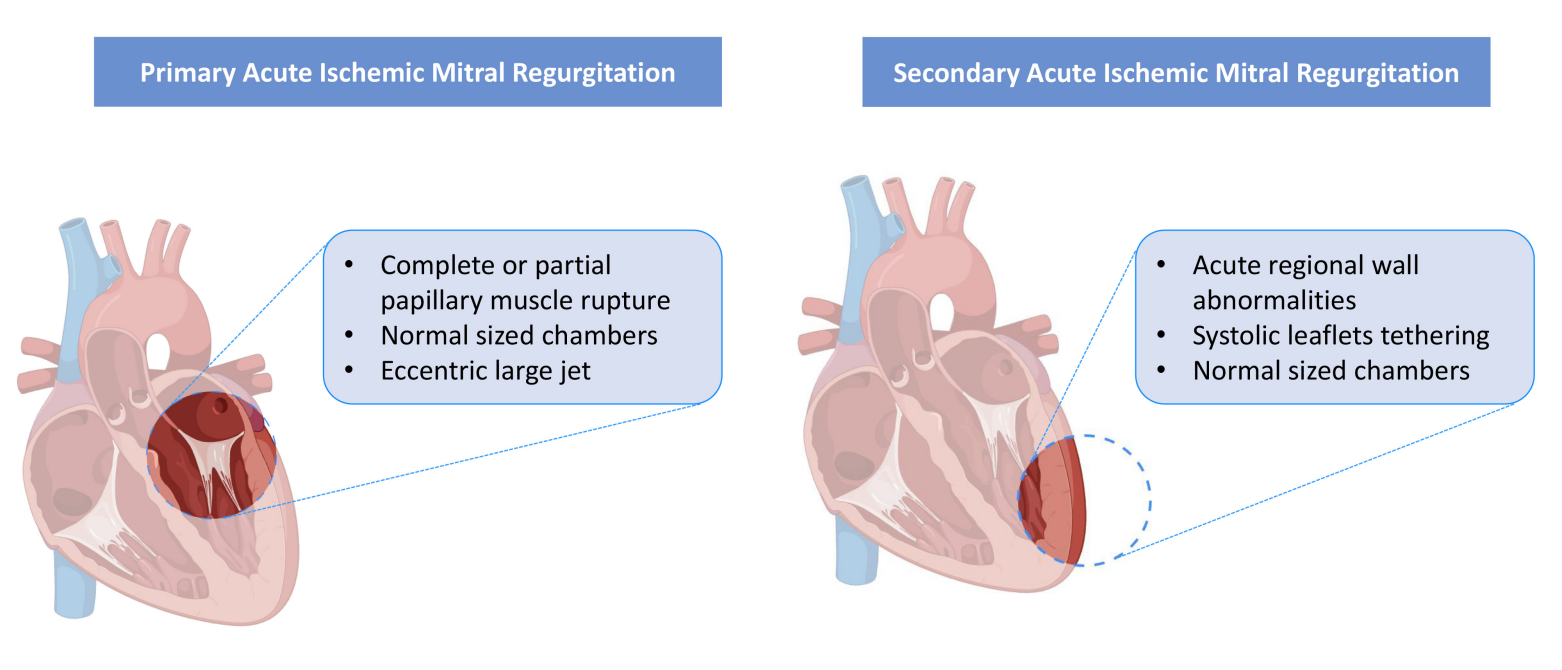
**Mechanism of acute mitral regurgitation for primary and secondary phenotype**.

The spectrum of PMR varies from simple elongation of the papillary muscle without rupture to partial rupture of one of the heads and, in severe cases, complete rupture. Complete PMR triggers torrential MR, acute pulmonary edema, and hemodynamic collapse, necessitating emergency intervention (Fig. 1) [4, 5].

Unlike the anterolateral papillary muscle, which benefits from a dual arterial circulation supply from the left anterior descending and the circumflex artery, the posteromedial muscle relies on a single circulation blood supply from the right coronary artery or circumflex artery, making it more frequent be involved [7].

AMR profoundly disrupts hemodynamic stability. When multiple chordae tendineae or a papillary muscle rupture, the left atrium and ventricle experience sudden volume overload, causing rapid hemodynamic decline and potentially leading to cardiogenic shock [8].

The acute management of this population necessitates hemodynamic stabilization and treatment of pulmonary edema. In these patients, especially with complete PMR, the role of revascularization is often not beneficial due to the severity of the condition. Therefore, surgical intervention remains the cornerstone therapy for patients with severe primary MR secondary to PMR; however, the surgical risk may be prohibitively high for some patients [5].

A systematic review of the literature consolidated evidence on transcatheter interventions for post-AMI PMR as a potentially viable alternative to surgery. This analysis showed that M-TEER was found to be a feasible approach, achieving “acceptable” device success in all cases [7]. Finally, it is worth emphasizing that in this population M-TEER can serve as a bridge to surgery. Stabilizing patients with M-TEER may allow for subsequent mitral valve replacement (MVR) in more favorable conditions rather than considering M-TEER solely as a definitive procedure.

### 2.2 Secondary Acute Ischemic Mitral Regurgitation

This type is also defined as AMR without sub-valvular apparatus rupture or functional AMR. It can occur in patients shortly after AMI, typically within one week of the initial event. Risk factors for MR are advanced age, comorbidities, female sex, non-smoking status, a higher Killip class (III or IV), and reduced left ventricle ejection fraction (LVEF) [9]. In this form of acute post-ischemic MR, the primary underlying mechanism involves altered ventricular geometry and displacement of the papillary muscles, resulting in inadequate coaptation of the mitral valve leaflets during systole. Despite retaining dynamic properties, the mitral apparatus exhibits leaflet separation and excessive angulation of the papillary muscles, leading to deformation of the mitral valve; the leaflets are structurally normal, but there is a reduced leaflet area-to-annular ratio. This mechanism is likely driven by sudden regional wall motion abnormalities, rather than significant global left ventricular remodeling (Fig. 1) [10]. In the acute phase, abnormal leaflet adaptation may result in severe MR, even with minor leaflet tethering [11]. Patients in this context can benefit from early revascularization, particularly concerning MR severity. A shorter time from symptom onset to reperfusion has been identified as a predictor of MR reduction in the early phase, due to alleviation of ischemia and preservation of myocardium, which influences subsequent left ventricular remodeling. Serial echocardiography often reveals fluctuating MR severity following AMI, especially after interventions such as primary PCI. Immediate changes in MR severity are commonly associated with early improvements in left ventricular function, while chronic changes reflect ongoing left ventricular remodeling or reverse remodelling [11]. While leaflet tethering due to papillary muscle displacement is typically observed in inferior-posterior AMI, it has also been documented in anterior AMI. In a retrospective study by Yosefy *et al*. [12], antero-apical AMI involving all apical segments was shown to cause papillary muscle displacement, leading to MR even in the absence of basal and mid-inferior wall motion abnormalities. Clinical outcomes in patients with functional MR are generally worse following anterior-wall AMI [13]. This disparity in outcomes is likely attributable to apical tethering of the leaflets and greater mitral annular deformation observed in anterior AMI, which is often associated with more severe left ventricular dysfunction [14].

## 3. Prevalence, Prognosis and Imaging

The prevalence of AMR after AMI varies across the studies. This is strictly related to different periods when such studies were performed, some in the fibrinolysis era and others in the primary PCI era. Additionally, the timing to assess the MR after the patient index admission varies between studies [1, 2, 9, 11, 15].

A recent study, involving 1000 patients, observed the onset of MR after primary PCI in 29% (n = 294) of the population, of whom 76% of the cases were mild, 21% moderate, and 3% severe [1]. Additionally, the MI subtype (ST-elevation myocardial infarction (STEMI) or non-ST-elevation myocardial infarction (NSTEMI)) did not affect MR prevalence despite the timing of the revascularization. After a mean follow-up of 3.2 years, all-cause mortality amongst patients without MR was 6% vs 19% in patients with MR, considering every grade (*p* < 0.001). An additional analysis focused on comparing mild MR and no MR groups reported a significantly higher all-cause mortality amongst patients with mild MR (37 of 220 [17%] vs 45 of 706 [6%]; *p* < 0.001, respectively). It underscores how the presence of MR by itself is sufficient to worsen long-term survival [1].

Another retrospective study which included 4005 STEMI patients undergoing primary PCI showed that none, 1+, 2+, 3+, and 4+ MR was diagnosed in 3200 (79.9%), 427 (10.7%), 260 (6.5%), 91 (2.3%), and 27 (0.7%) patients, respectively, The 5-year follow-up showed higher mortality rates for each grade of MR [none = 16.2%, 1+ = 23.1%, 2+ = 36.5%, 3+ = 53.8%, and 4+ = 63% (*p* < 0.001)]. Similarly to the above, the presence of mild MR after AMI was associated with a 6.9% increase in mortality compared to none MR group [2].

Across the studies, the identified risk factors for severe AMR after AMI include age, female gender, heart failure, multivessel disease, timing of revascularization, and left ventricular dysfunction [1, 5, 16].

The diagnosis of AMR is not always straightforward, especially for the secondary phenotype, because the clinical picture is frequently ambiguous and it may develop gradually over the course of several days, thus, the high clinical suspicion is essential in avoiding diagnostic delays. The presence of acute heart failure after AMI and a new systolic murmur should raise the suspicion, excluding AMR as a possible cause.

Despite chronic MR, the diagnosis of AMR can be difficult for different reasons such as the poor transthoracic window caused by pulmonary edema and the underestimation of AMR severity in patients with high LA pressure and low blood pressure, which causes the reduction of left ventricle power and the regurgitation jet across the valve at color Doppler imaging evaluation. Additionally, the quantitative parameters to grade the severity of the MR are only approved for chronic MR.

Nevertheless, echocardiographic assessment is paramount to confirm the diagnosis and understand the underlying mechanism.

In patients post AMI and acute onset of dyspnea the performance of transthoracic echocardiography (TTE) should be the first-line evaluation.

TTE enables a full assessment of the LVEF, dimensions and regional wall abnormalities, the mitral valve apparatus, and MR estimation. The finding of a papillary muscle rupture or flail leaflet in a patient with pulmonary edema after AMI is enough to establish the diagnosis even in the absence of a large MR jet showed by the color Doppler. In the case of secondary AMR, caused by acute ischemic regional wall abnormalities, the leaflets appear anatomically normal with the presence of tethering. Notably, in comparison to chronic MR, the acute onset of severe MR could be detected despite the small leaflet tethering caused by the failure of the leaflet adaptation [17].

In case of a scarce echocardiographic window with poor visualization, transesophageal echocardiography (TEE) should be performed to establish the diagnosis and assess the severity of the regurgitation [18]. TEE is essential in candidates for intervention, allowing for a more complete valvular evaluation and the following decision between surgery or transcatheter intervention.

An integrative approach incorporating qualitative, semiquantitative, and quantitative parameters is advised.

## 4. Revascularization and Mechanic Circulatory Support(MCS)

**Fig. 2.**
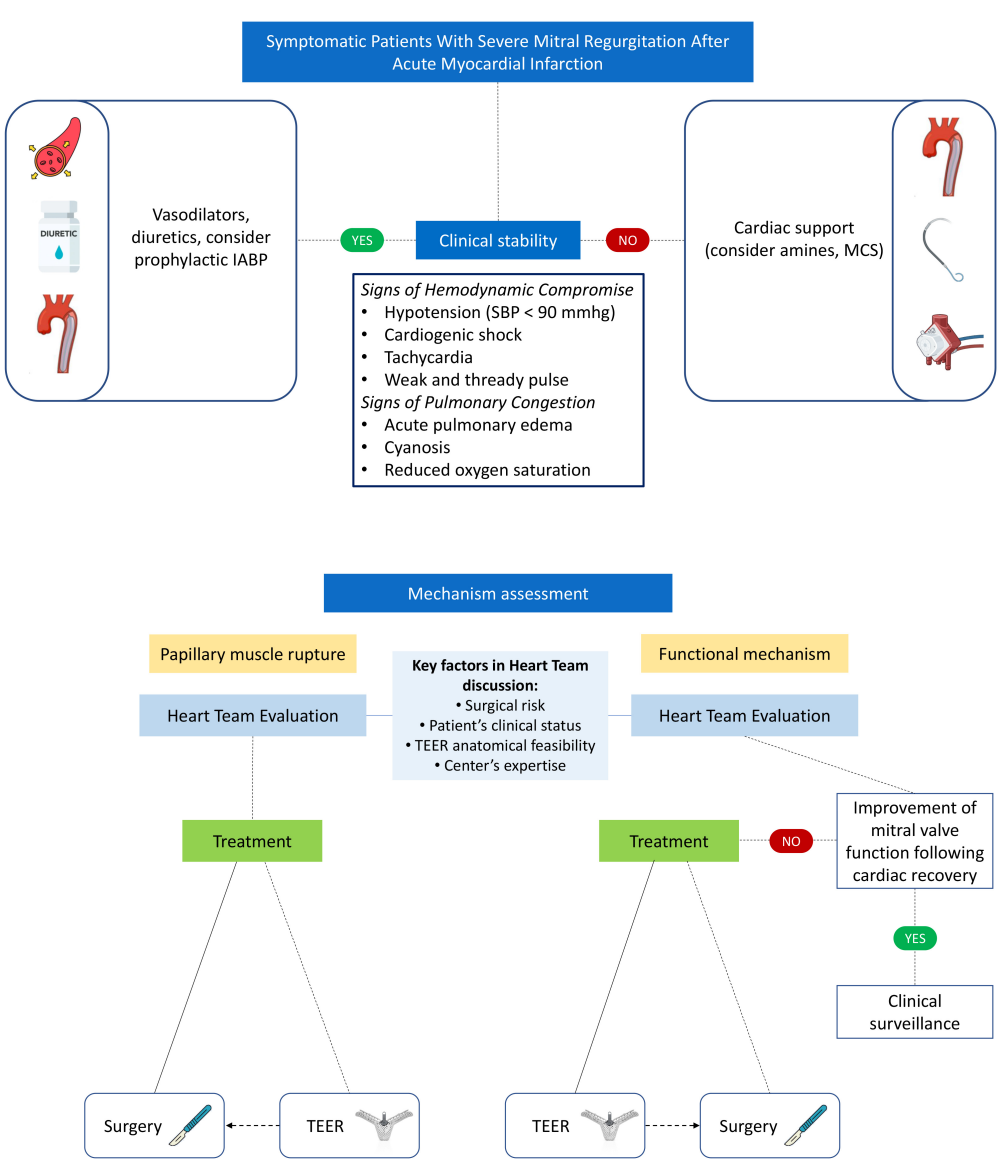
**Management of severe mitral regurgitation after acute myocardial infarction according to clinical patient stability and mitral regurgitation mechanism assessment**. IABP, intra-aortic balloon pump; MCS, mechanical circulatory support; TEER, transcatheter edge-to-edge repair; SBP, systolic blood pressure.

A rapid and successful primary PCI is the mainstay to prevent the onset of AMR and should be the immediate intervention in the absence of PMR [11]. It showed to acutely reduce the degree of MR [11]. For this reason, patients with unsuccessful reperfusion or late acute coronary syndrome presentation may require special attention and further intervention. Nevertheless, PMR frequently results in acute hemodynamic deterioration or CS despite a successful primary PCI [18]. In this setting, apart from the role of surgical or transcatheter treatment that will be discussed in the next paragraphs, the impact of MCS as a bridge to intervention and the post-procedural period is becoming increasingly important (Fig. 2).

The use of MCS can stabilize patients with CS before, during and after intervention and, thus, potentially reduce the mortality rates in this population. The available data on MCS as a bridge to surgery seems to show a tendency to improve peri-procedurally hemodynamic parameters and post-procedural outcomes, but these evidences are limited by the small number of patients included [19, 20].

The current guidelines recommend the use of intra-aortic balloon pump as first-line cardiocirculatory support therapy in patients with ischemic AMR [21]. In these populations intra-aortic balloon pump is not always sufficient to guarantee an adequate perfusion. In cases of refractory CS the use of intravascular microaxial flow pump devices, like Impella CP or Impella 5.5 (Abiomed), represent a powerful MCS and the next step in the management of such patients. The last resort in more severe cases is the implantation of femoral venoarterial extracorporeal membrane oxygenation (VA-ECMO or TandemHeart). The VA-ECMO increases the left ventricle afterload and filling pressure, preventing myocardial recovery and potentially exacerbating pulmonary edema, especially in patients with reduced LVEF at admission. In that scenario, the concomitant use of Impella (ECMELLA) should be considered to reduce left ventricle filling pressure and improve cardiac function.

The emerging role of MCS in the management of this population is essential to reduce the high hospital mortality that has not substantially changed in the last decades.

## 5. Surgical Treatment of Acute Ischemic Mitral Regurgitation

### 5.1 Indication and Timing of Surgery

Surgery is still the main invasive treatment used in patients who develop severe MR shortly after AMI and, before the introduction of transcatheter therapies, it was the standard and only approach.

Indication and timing of surgery are usually defined by well-established clinical and echocardiographic factors: degree of MR, LV function and dimension, pulmonary artery systolic blood pressure, presence of atrial fibrillation, clinical symptoms, patient comorbidities, and response to medical therapy [21]. Moreover, the mechanism underlying MR plays a key role. Severe mitral regurgitation due to complete or partial papillary muscle rupture following myocardial infarction often necessitates urgent mitral valve surgery [5]. Indeed, this condition commonly leads to severe hemodynamic instability and potentially to cardiogenic shock, so prompt surgical treatment is needed to increase the odds of survival [22]. When stabilization of the patient’s hemodynamic status is achieved, surgery could be postponed by a few days [23]. A delayed surgical approach permits fibrotic tissue maturation and is tied to better surgical outcomes, mainly in patients without early hemodynamic compromise or shock criteria [24]. Nevertheless, in-hospital mortality in this setting is still high ranging between 25 and 40% but the mortality rate without intervention can reach 80% [25, 26].

On the other hand, in the case of acute post-ischemic functional MR, medical treatment is the first-line treatment, and surgery is considered if symptoms persist [27]. In this context, particularly in patients with reduced LVEF, surgery plays a less prominent role than PMR, making M-TEER the potential preferred treatment option as occurred in the last decade in the management of chronic functional MR.

### 5.2 Mitral Valve Repair vs Replacement

Randomized trials and observational multicenter studies comparing mitral valve repair vs replacement in ischemic MR, failed to show any difference in terms of LV remodeling or survival after 1 and 2 years, with however a higher MR recurrence rate and cardiac-related hospital readmission in the repair group [5, 28].

Nevertheless, for patients with acute ischemic MR, international guidelines recommend, whenever possible, the use of mitral valve repair instead of replacement [27].

Mitral valve repair is a viable approach in the case of functional post-ischemic MR, and several techniques have been adopted in this setting (e.g., annuloplasty, papillary muscle approximation, or edge-to-edge technique) [5]. In cases with complete PMR, mitral valve repair is often not feasible because of necrotic and friable infarcted tissue, and MVR should be considered [5].

Even though not indicated as the first approach by current guidelines, MVR is more commonly performed in patients with acute ischemic MR because of its greater reproducibility and durability [6]. Moreover, its use in patients with refractory CS is strongly recommended [26]. If MVR is performed, preserving the subvalvular apparatus and so the mitral-ventricular continuity could guarantee better long-term survival [29].

## 6. Mitral Transcatheter Edge-To-Edge Repair

In the last decade, M-TEER has been established as a valid option for open heart surgery in patients with chronic severe MR [27, 30]. To date, CE and Food and Drug Administration (FDA)-approved devices to perform M-TEER are Mitraclip (Abbott Vascular) and Pascal (Edwards Lifesciences). These systems have different features but proved similar effectiveness, improving quality of life, symptoms and survival [31, 32, 33, 34, 35, 36].

**Fig. 3.**
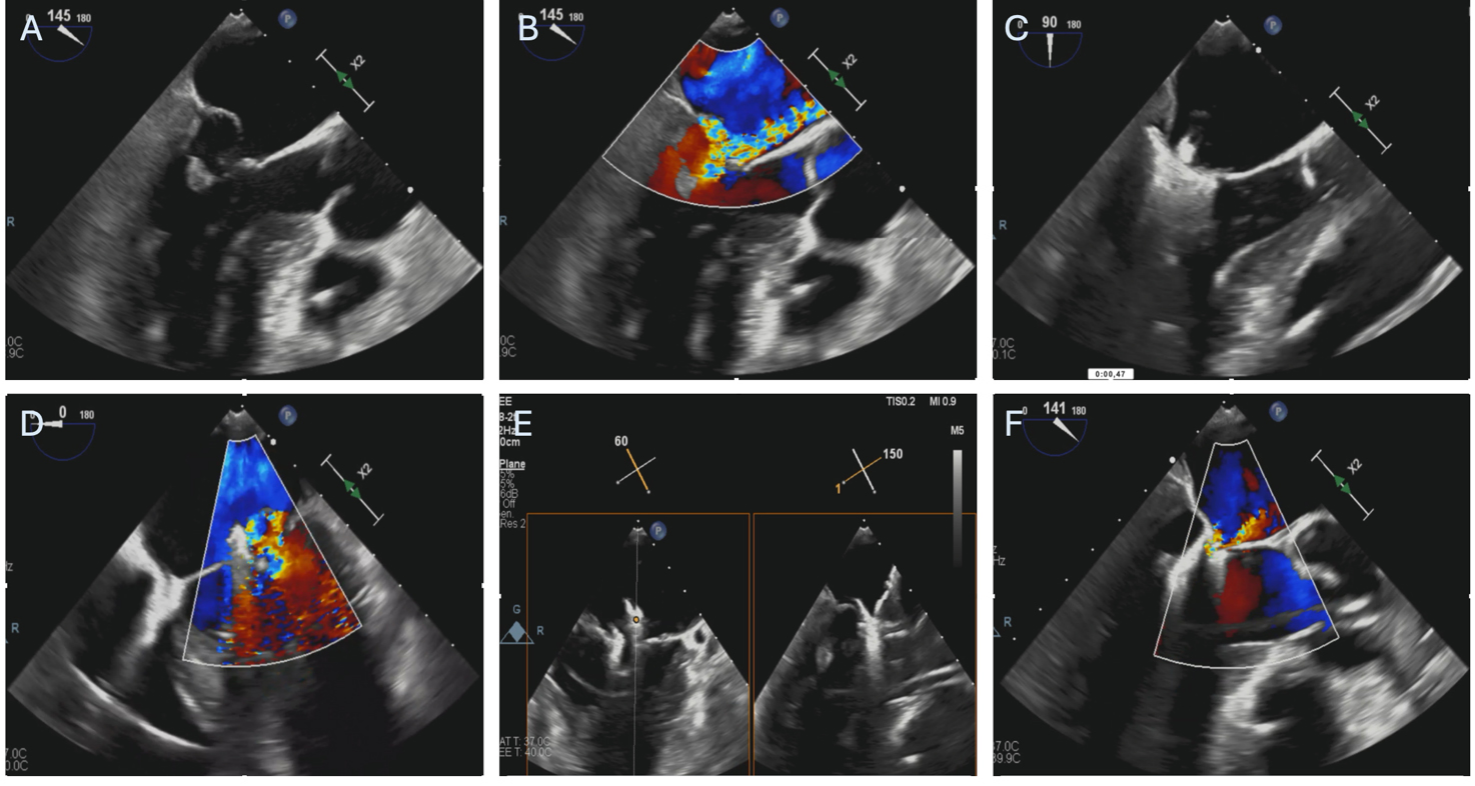
**Patient with severe mitral regurgitation after myocardial infarction treated with M-TEER**. (A,B) Severe mitral regurgitation due to anterolateral papillary muscle rupture and subsequent P2 leaflet flail. (C) First Mitraclip XTW positioning, before grasping. (D) Result after releasing the first Mitraclip XTW. (E) Orientation of the second Mitraclip XTW. (F) Final result after releasing the second. M-TEER, mitral transcatheter edge-to-edge repair.

Current evidence for the treatment of ischemic AMR is still limited, but much of it comes from the use of the Mitraclip system. An example of M-TEER performed in a patient with AMR caused by PMR after AMI is represented in Fig. 3.

The first data on feasibility of the transcatheter approach in this population stem from some case reports or case series in patients with PMR and at high risk for surgery, employing the Mitraclip system as a last resort (Table 1, Ref. [37, 38, 39, 40, 41, 42, 43, 44, 45, 46]) [47, 48, 49, 50]. Nowadays, after the description of these initial reports, M-TEER is a viable option in PMR after AMI. The procedure reduces MR, improving hemodynamic parameters and symptoms. The current recommendations, thus, encourage M-TEER in selected patients at prohibitive risk [6, 30]. Notwithstanding, the high rates of non-surgical candidates for such deteriorating condition makes this population an ideal substrate for M-TEER.

**Table 1.** **Studies evaluating the role of M-TEER in patients with severe mitral regurgitation after acute myocardial infarction**.

First author	Year	Patients	Mechanism of acute MR	Procedural outcomes	Follow-up
Estévez-Loureiro R. [41]	2015	N = 5	Functional	Device success = 100%	317 days
		Mean age = 68 yrs		In hospital mortality = 1/5, 20%	Mortality = 20%
					NYHA I/II = 4/5; 80%
					MR ≤2 = 4/5; 80%
Adamo M. [42]	2017	N = 5	Functional	Procedural success = 100%	2 years
		Mean age = 73 yrs		In hospital mortality = 0/5, 0%	Mortality = 2/5; 40%
		Male (60%)			NYHA I/II = 2/3; 66.6%
					MR ≤2 = 2/3; 66.6%
Haberman D. [43]	2019	N = 20	Functional	Procedural success = 95%	15 months
		Mean age = 68.1 yrs		30-days mortality = 1/20, 5%	Mortality = 2/20; 10%
		Male (30%)			NYHA I/II = 17/20; 85%
					MR ≤2 = 16/20; 80%
Estevez-Loureiro R. [38]	2020	N = 44 patients	Functional	Technical success = 86.6%	6 months
		Mean age = 70 yrs		30-day mortality = 9.1%	Mortality = 18.2%
		Male (64%)			NYHA I/II = 75.9%
					MR ≤2 = 72.5%
Jung R.G. [39]	2021	N = 141 patients	Functional = 106	In hospital mortality = 15.6%	1 year
		Mean age = 68.9 yrs	PMR = 33		Mortality = 42.6%
		Male (55%)	Both = 2		NYHA I/II = NA
					MR ≤2 = 88.7%
Haberman D. [44]	2021	N = 105	Functional = 99	Procedural success = 91.4%	1 year
		Mean age = 70.5 yrs	PMR = 6	In hospital mortality = 9%	Mortality = 15.2%
		Male (52%)			NYHA I/II (LVEF <35) = 54%
					NYHA I/II (LVEF ≥35) = 68%
					MR ≤2 (LVEF <35) = 71%
					MR ≤2 (LVEF ≥35) = 76%
So C.Y. [45]	2022	N = 24 patients	PMR	Device success = 68.8%	30 days
		Mean age = 73 yrs		30-days all-cause mortality = 9%	Mortality = 2/22; 9.1%
		Male (71%)			NYHA I/II = NA
					MR ≤2 = NA
Chang C.W. [46]	2022	N = 5 patients	PMR	Device success = 100%	30 days
		Mean age = 75 yrs		In hospital mortality = 4/5, 80%	Mortality = 4/5, 80%
		Male (40%)			NYHA I/II = 1/1
					MR ≤2 = 1/1
Haberman D. (IREMMI Registry) [40]	2022	N = 99 patients	Both functional and PMR	Procedural success = 93%	1 year
		Mean age = 71 yrs		In hospital mortality = 6%	Mortality = 17%
		Male (48%)			NYHA I/II = 61%
					MR ≤2 = 66%
Haberman D. [37]	2024	N = 23	PMR	Acute Procedural success = 87%	1 year
		Mean age = 68 yrs		In hospital mortality = 30%	Mortality = 34.7%
		Male (56%)			NYHA I/II = 69%
					MR ≤2 = NA

Abbreviations: N, number of patients; MR, mitral regurgitation; NYHA, New York Heart Association; PMR, papillary muscle rupture; yrs, years; NA, not available.

The main contribution in the field of M-TEER and acute post-ischemic PMR is a sub-analysis of the international, multicenter IREMMI registry including 23 patients of whom 9 patients had complete papillary muscle rupture, 9 partial and 5 chordal rupture [37].

Thirteen patients (57%) presented with STEMI, of whom 10 (70%) suffered inferior AMI and 7 (30%) had an anterior AMI. The patients were refused surgery because deemed at prohibitive risk (median Euroscore II = 27%) and 87% (n = 20) were in CS. Vasopressors were administered to all subjects and mechanical support was used in 74% of the cases (11 intra-aortic balloon pump (IABP), 2 Impella, and 4 VA-ECMO). The median LVEF was 45% and M-TEER was performed on a median 6 days after AMI. The rate of acute procedural success was 87%, resulting in rapid hemodynamic improvement with a significant reduction in left atrial pressure and systolic pulmonary artery pressure.

In-hospital mortality was 30%, an acceptable rate considering the severity of the condition and the absolute contraindication for surgery, and 22% of the patients at follow-up underwent elective, successful surgical MVR. This underscores the potential impact of M-TEER in the clinical stabilization of the patient before an eventual surgical intervention in a more stable clinical setting.

Finally, the device used in the present study is the second generation of the Mitraclip system, and therefore with the commercially available latest iteration device (Mitraclip G4, fourth generation), the procedural results and repair durability should further improve [51].

Concerning the acute post-ischemic functional MR, the actual available data are promising for the M-TEER approach in this setting.

A multicenter registry analyzed 44 patients with prohibitive surgical risk subjects (median Euroscore II = 15.1%) who underwent M-TEER after a mean time 18 days after AMI [38]. Twenty-eight patients (63.7%) were diagnosed with NYHA functional class IV. The rate of technical success was reached in 86.6% of the cases and the mortality at 30 days was 9.1%. At median follow-up of 4 months, 75.9% of the patients were in NYHA functional class I to II and mild to moderate degree of MR was noted in 72.5% and was observed in 75.9% of survivors [38].

The role of M-TEER in patients with functional AMR and CS was assessed in the multicenter IREMMI registry which enrolled 95 subjects [52]. Patients with CS were younger (68 ± 10 vs 72 ± 9 years, respectively) and had high surgical risk (21 ± 18% vs 11 ± 8% Euroscore II; *p* = 0.001) compared to those without CS. The use of vasoactive drugs (82% vs 4%, *p* < 0.001) and MCS (IABP/Impella, 66% vs 7%, *p* < 0.001) or VA-ECMO (12% vs 0%, *p* = 0.028) were more frequent in the CS group. Technical success was 100% and the immediate procedural success was 90% and 93% in CS and non-CS groups, respectively. At 30-day follow-up, the mortality rate was 10% and 2.3% in CS and non-CS, not reaching statistical difference. At 3 months, NYHA functional class and degree of MR ≤2 (83.4% CS vs 90.5% non-CS, *p* = 0.348) were consistently improved, without difference between groups. Likewise, the combined rate of mortality and heart failure re-hospitalization, at a median follow-up of 7 months, was comparable (28% CS vs 25.6% non-CS; *p* = 0.793).

Subsequently, other studies showed favorable results in CS patients treated with M-TEER [39, 53].

The larger multicenter study published in the field of post-ischemic AMR and M-TEER comprised 471 patients and excluded those with PMR from the analysis for different prognoses and often treated with urgent intervention [40].

The overall population received conservative treatment and intervention in 56% (n = 266) and 44% (n = 205), respectively. In the intervention group, 52% (n = 106) of the patients underwent surgery, whilst 48% (n = 99) M-TEER. Among those surgically treated, 60 (57%) underwent MVR and 45 (43%) mitral valve repair. Patients treated with the transcatheter approach presented more severe clinical conditions because 52% of them had cardiogenic shock, in comparison with 31% in the surgical group (*p* < 0.01) and had lower LVEF (35% ± 11% vs 45% ± 10%, *p* < 0.01). Patients in the conservative group experienced the worst outcomes.

In the surgical cohort, in-hospital mortality was significantly higher compared to the M-TEER group (16% vs 6%, *p* < 0.01). After a median follow-up of 239 days, using propensity matching score adjustment, the mortality rate was higher in patients surgically treated compared with those undergoing M-TEER, mainly driven by in-hospital mortality.

To date, the available evidence in the field comes from observational studies, most them with small sample size, and results of some randomized trials are expected to better understand and address the role of M-TEER in this population. It is also important to highlight that considered the severity of the condition, the conduction of clinical trials are challenging in terms of recruitment and management. Nevertheless, the transcatheter approach is undoubtedly an additional weapon in our therapeutic arsenal and should be used with caution by experienced operators in this setting.

The analysis and summary of the evidence discussed so far leads us to a practical approach for the management of this population.

First of all, it is crucial to emphasize again the paramount contribution of the myocardial revascularization for patients’ outcomes.

In patients with PMR the first therapeutic step should be the medical therapy (diuretics, vasodilators) or preventive IABP placement in case of hemodynamic stability and the use of MCS if hemodynamic instability is present. Subsequently, considered the catastrophic prognosis without invasive treatment, mitral surgery should be offered if the subject is a good surgical candidate. In case of contraindication to surgery, the patient should be treated with transcatheter approach.

In patients with functional AMR, medical therapy plays an important role and may be sufficient to stabilize the clinical picture. If not, the placement of MCS can be helpful for improving the hemodynamics. In case the clinical scenario requires an invasive treatment, the upfront treatment with M-TEER is suggested in expert center or the patients can undergo MVR, if the surgical risk is not prohibitive. In both scenario of functional AMR or PMR, M-TEER can be an option to stabilize the clinical condition as a bridge to the definitive surgery when the surgical risk for the patient will be significantly lower (Fig. 2).

## 7. Ongoing Issues and Future Perspectives

M-TEER procedure post-AMI is still an emerging field, and various uncertainties must be addressed.

First, the correct timing for intervention is unclear. The average time between AMI and M-TEER, in the analyzed studies, is 20 days and an earlier procedure could improve the outcomes. Second, it is unclear which clinical and anatomical criteria should be assessed to select an ideal candidate for M-TEER. Third, the transcatheter repair durability in this population is uncertain. Additionally, M-TEER precludes future surgical repairs, offering only the possibility of surgical replacement. Fourth, M-TEER in this setting was performed as a rescue approach, thus, it is unknown the potential role in patients with mild symptoms in order to prevent the catastrophic consequences of post-ischemic acute MR. Fifth, the role of a different device like the Pascal system, in this setting, is still to be assessed. Finally, an in-depth evaluation of the concomitant use of MCS and M-TEER, in this high-risk population, is crucial because could be the game changer for improving in-hospital mortality and long-term outcomes.

## 8. Conclusion

M-TEER procedure with Mitraclip device in acute post-ischemic MR showed to be a safe and effective approach and should be present in all algorithms for the management of AMR.

However, more studies and randomized clinical trials are needed to fully understand short and long-term outcomes. The EMCAMI Trial (Early Mitral Valve Repair After Myocardial Infarction, NCT06282042) will assess the safety and efficacy of Mitraclip device in clinically significant functional MR within 60 days after acute myocardial infarction and without cardiogenic shock compared to standard of care. On the other hand, the CAPITOL MINOS Trial (Evaluating the role of mitral interventions post-MI, NCT05298124) will randomize patients with Society for Cardiovascular Angiography & Interventions (SCAI) stage C and D cardiogenic shock, SCAI stage C or D cardiogenic shock, with persistent inotrope/vasopressor/non-durable mechanical support or unable to wean ventilatory support due to pulmonary edema for 24 hours prior to randomization, to M-TEER or standard of care. These two trials will be able to cover the entire clinical spectrum of severity of the post-AMI MR and shed lights on the role of M-TEER in this rare but catastrophic disease.

Finally, a multidisciplinary heart team is the cornerstone to offer the best and timely treatment.
